# PHB+aPHA Blends: From Polymer Bacterial Synthesis through Blend Preparation to Final Processing by Extrusion for Sustainable Materials Design

**DOI:** 10.3390/ma17133105

**Published:** 2024-06-25

**Authors:** Tomasz M. Majka, Konstantinos N. Raftopoulos, Edyta Hebda, Adam Szeligowski, Olga Zastawny, Maciej Guzik, Krzysztof Pielichowski

**Affiliations:** 1 Department of Chemistry and Technology of Polymers, Cracow University of Technology, ul. Warszawska 24, 31-155 Kraków, Poland; 2 Jerzy Haber Institute of Catalysis and Surface Chemistry, Polish Academy of Sciences, ul. Niezapominajek 8, 30-239 Kraków, Poland; 3 ORLEN Południe S.A., Fabryczna 22, 32-540 Trzebinia, Poland

**Keywords:** biopolymer blends, polyhydroxyalkanoate (PHA), polyhydroxybutyrate (PHB), processing, sustainable materials, brittleness, thermal and mechanical properties

## Abstract

The inherent brittleness of polyhydroxybutyrate (PHB), a well-studied polyhydroxyalkanoate (PHA), limits its applicability in flexible and impact-resistant applications. This study explores the potential of blending PHB with a different PHA to overcome brittleness. The synthesis of PHA polymers, including PHB and an amorphous medium-chain-length PHA (aPHA) consisting of various monomers, was achieved in previous works through canola oil fermentation. Detailed characterization of aPHA revealed its amorphous nature, as well as good thermal stability and shear thinning behavior. The blending process was carried out at different mass ratios of aPHA and PHB, and the resulting blends were studied by differential scanning calorimetry (DSC), X-ray diffraction (XRD), scanning electron microscopy (SEM), and thermogravimetric analysis (TGA). The blends exhibited complex DSC curves, indicating the presence of multiple crystalline forms of PHB. SEM images revealed the morphology of the blends, with PHB particles dispersed within the aPHA matrix. TGA showed similar thermal degradation patterns for the blends, with the residue content decreasing as the PHB content increased. The crystallinity of the blends was influenced by the PHB content, with higher PHB ratios resulting in an increased degree of crystallinity. XRD confirmed the presence of both α and β crystals of PHB in the blends. Overall, the results demonstrate the potential of PHB+aPHA blends to enhance the mechanical properties of biopolymer materials, without com-promising the thermal stability, paving the way for sustainable material design and novel application areas.

## 1. Introduction

Among biopolymers, polyhydroxyalkanoates (PHA) have emerged as a versatile class with vast potential for applications in sectors such as packaging, biomedical devices, and agriculture [1]. One of the most extensively studied polymers within this class is polyhydroxybutyrate (PHB). PHAs exhibit numerous desirable properties, including biocompatibility, biodegradability, and thermo-plasticity, making them an attractive choice for a wide range of industries [2]. In packaging, PHA films can replace conventional plastic films, offering sustainable and biodegradable alternatives [3]. Moreover, PHA’s biocompatibility makes it suitable for biomedical applications, such as drug delivery systems, tissue engineering scaffolds, and sutures [4,5,6]. In the agricultural sector, PHA can be used as a coating material for controlled-release fertilizers, mulch films, and seed encapsulation, contributing to sustainable farming practices [7].

Despite their promising attributes, such as biodegradability and renewability, polyhydroxyalkanoates face limitations that hinder their broader adoption across industries. One notable limitation is the inherent brittleness of polyhydroxybutyrate, which restricts its use in applications requiring flexibility and impact resistance. Overcoming this constraint necessitates the exploration of innovative solutions [8].

The commonly used poly[(R)-3-hydroxybutyrate] (PHB) is a highly crystalline material, which provides it with mechanical strength but also makes it brittle [9,10]. Disadvantages include limited thermal stability, which can be improved by blending with other biopolymers or by copolymerization with, for example, polyhydroxybutyrate [11,12]. However, as reported in the literature, such attempts lead to a significant reduction in the degree of crystallinity of the copolymer, resulting in thermoplastic elastomers [13,14]. The melting point of PHB is about 175 °C, and for this reason, it can be successfully used as a substitute for polypropylene, for example, in the packaging industry [15,16,17]. Regardless of whether PHB crystallizes from a melt or a solution, its α form is usually obtained. The β form is generally formed by stretching PHB films and fibers and is a less stable and less ordered form than the α form [18,19,20].

The high production costs associated with PHAs have posed significant challenges to their commercial viability. The complex and resource-intensive manufacturing processes, along with the limited scalability of PHA production, have contributed to these high costs [21,22]. Consequently, finding cost-effective methods for large-scale PHA production has become a crucial focus of research and development. Therefore, such an approach to implement biorefining has gained more attention in recent years. In our previous study, we successfully demonstrated the efficient production of PHA polymers within a biorefinery process utilizing canola oil [23]. Through hydrolysis of this substate, two distinct fractions for fermentation were obtained: fatty acids and glycerol, which give rise to two different PHA polymers—amorphous PHA (aPHA) and PHB [24,25]. The amorphous PHA is a medium-chain-length PHA consisting of various hydroxyalcanoate monomers [25,26].

The thermal and mechanical properties of the PHB+aPHA blends were evaluated and compared to other polymer materials currently used in similar applications. The thermal degradation temperature (T_deg_) of PHB+aPHA blends is consistent with other PHA polymers but is approximately 40 °C lower than that of PE [27] and PLA [28]. The glass transition temperature (T_g_) of the blends aligns with the T_g_ of aPHA, which is significant as other PHA elastomers like PHO [29] have a T_g_ close to that of PE (−30 to −35 °C) [30]. By varying the aPHA content in the blend, the degree of crystallinity can be modified to match the properties of PE (approximately 36%) [31] or PLA (35–45%) [32].

Although the mechanical properties were not the primary objective of this study, it is important to note that PHB typically has a high Young’s modulus (600–3500 MPa, depending on the PHB type) [33]. The addition of aPHA can reduce this modulus; for instance, PHO, another PHA, exhibits a Young’s modulus of approximately 14–15 MPa [29]. Future research could focus on other mechanical tests, such as elongation at break and fatigue tests, to further understand the potential applications of these blends. This study highlights the versatility of PHB+aPHA blends and their potential to meet the requirements of various applications through careful adjustment of their composition and processing conditions.

The biodegradation of PHB+aPHA blends, including both hydrolytic and enzymatic degradation, is indeed an interesting and relevant topic for the readers. Based on previous research, it was observed that polyhydroxyalkanoates (PHAs), such as PHB and its blends, exhibit significant biodegradability under various conditions [34]. In particular, PHB demonstrates complete degradation in environments such as marine pelagic, freshwater aerobic, and aquatic mesophilic anaerobic conditions. The incorporation of amorphous PHA (aPHA) into PHB can potentially influence these biodegradation properties, making the blend more adaptable to different environmental conditions. In the context of hydrolytic and enzymatic degradation, the cited work highlights that PHAs can be broken down by microbial action, leading to the production of biogas, primarily methane, under anaerobic conditions. This suggests that PHB+aPHA blends could also exhibit enhanced biodegradability, producing valuable biogas as a byproduct. Furthermore, blends containing PHB have shown faster degradation rates under industrial composting conditions compared to their individual polymer components. To address this question comprehensively, we plan future studies focusing specifically on the hydrolytic and enzymatic degradation of PHB+aPHA blends. Such studies could involve detailed analysis of biodegradation products under controlled laboratory conditions as well as real-world environmental scenarios to evaluate the biodegradation kinetics and mechanisms involved. This would provide deeper insights into the environmental impact and potential applications of these biopolymer blends in sustainable materials design.

Blending these two types of PHAs offers a promising approach to overcoming the limitations associated with pure PHB and achieving customized properties for specific applications. The combination of aPHA and PHB can significantly enhance the mechanical properties, provide higher flexibility, and improve the overall performance of the resulting biopolymer blends. Thus, this study aims to comprehensively investigate the physicochemical and thermal characteristics of blends comprising amorphous polyhydroxyalkanoate and polyhydroxybutyrate. Through an in-depth analysis of the blending process, polymer compatibility, and intermolecular interactions, valuable insights can be gained into the behavior and properties of aPHA+PHB blends. By elucidating the original structure–property relationships, this research contributes to the development of biopolymer blends with tailored properties for various applications.

Our research seeks to advance the field of biopolymer technology and facilitate the design of sustainable materials with enhanced performance characteristics. The findings from this study will provide valuable information for industries and researchers interested in utilizing biopolymers, particularly PHA and PHB blends, for a diverse range of applications. The knowledge gained will contribute to the scientific community’s understanding of polymer blends and aid in the development of eco-friendly materials that meet the stringent requirements of the modern world, furthering the progress towards a more sustainable future.

## 2. Materials

### 2.1. P(3HB) Synthesis

Poly(3-hydroxybutyrate) (P(3HB)) was produced by bacterial fermentation from glycerol obtained from canola oil hydrolysis at 42 °C using bacterial strain *Zobellella denitrificans* MW1 (Wilhelms-Universität Münster, Münster, Germany) at 200 L scale, as described previously [24]. Medium-chain-length PHA, an amorphous polymer (aPHA), was produced by bacterial fermentation from canola-oil-derived fatty acids at 30 °C using the bacterial strain *Pseudomonas putida* CA-3 (University College Dublin, Dublin, Ireland) at 200 L scale [25]. aPHA in this study consists of several monomers, primarily 3-hydroxyoctanoate (≈27 mol%), 3-hydroxynonanoate (≈24 mol%), 3-hydroxyheptanoate (≈17 mol%), and 3-hydroxydecanoate (≈16 mol%), and smaller amounts of shorter and longer monomers. For each of the biopolymers, the biomass after fermentation was lyophilized and then extracted with chloroform or ethyl acetate (Chempur, Piekary Śląskie, Poland), correspondingly. The resulting solution was filtered through activated charcoal (Merck, Warsaw, Poland) and a 0.2 µm polytetrafluoroethylene (PTFE) filter (Avantor, Gdańsk, Poland). Next, the polymer solution was concentrated on a rotatory evaporator (Heidolph Hei-VAP Industrial B, Heidolph Instruments GmbH & Co. KG, Schwabach, Germany), precipitated in an ice-cold methanol solution (Chempur, Piekary Śląskie, Poland), and dried in an oven (Binder FED400, Binder GmbH, Tuttlingen, Germany). The purified polymers were reconstituted in chloroform (5% *w*/*v*) for further experiments.

### 2.2. Solvent Casting (STEP I)

The sample preparation was conducted in two stages. The first stage (STEP I) involved preparing mixtures of aPHA and PHB in selected mass ratios by mixing and dissolving them in chloroform (Merck Life Science, Darmstadt, Germany) at a temperature of 55 °C. A magnetic stirrer WIGO MS 11 H (Wigo, Pruszków, Poland) was utilized for this purpose. The solvent-casting protocol, described in our patent application No. P.434423 “Method of crystallization of polyhydroxyoctanoate from solution” [35], was employed to obtain the blends. A series of blends was obtained, with PHB content 0, 50, 60, 70, 80, 90, and to 100 wt.%. Subsequently, the biopolymer blends were poured out of the solution, and the solvent was allowed to evaporate for 9 days. The blends are denoted as XX:YY, with XX being the mass fraction (wt.% of PHB) and YY that of aPHA. The 100 wt.% samples are denoted only by the name of the polymer.

### 2.3. Extrusion Process (STEP II)

For the extrusion of the selected PHB+aPHA blends obtained in the previous step, a Thermo Scientific HAAKE MiniCTW twin-screw mini-extruder (Thermo Scientific HAAKE, Poznań, Poland) was used under conditions previously selected for the polyhydroxyalkanoates processing. The extrusion was carried out at the temperature of two heating zones of 175 °C with the rotational speed of the screws at 50 rpm and in the torque range of 8–23 Nm. The PHB+aPHA blends obtained in Step I, which had the best physicochemical properties, were selected for processing in Step II (90:10, 70:30, 60:40). Similar to above, the blends are denoted as XX:YY, with XX being the mass fraction (wt.% of PHB) and YY that of aPHA. The 100 wt.% samples are denoted only by the name of the polymer.

## 3. Methods

### 3.1. X-ray Diffraction (XRD) Analysis

The XRD measurements were made on solvent-casted samples using a D2 Phaser X-ray powder diffractometer by Bruker. The copper lamp emitted radiation of 1.54 Å. Measurements were performed in the 2*θ* range of 1–40° using a 0.1 mm aperture and a 0.5 mm diaphragm.

### 3.2. Infrared Spectrometry (FTIR)

Fourier transform infrared measurements were performed using a Nicolet iS5 spectrometer (Thermo Fisher Scientific, Waltham, MA, USA) equipped with a diamond crystal attenuated total reflectance unit (ATR). Spectra were measured from 4000 to 400 cm^−1^, with an average of 16 scans.

### 3.3. Scanning Electron Microscopy (SEM)

Secondary electron scanning electron microscopy (SEM) micrographs were taken using a JEOL InTouchScope JSM-6010LV (JEOL Ltd., Tokyo, Japan) microscope, operated at 10 kV accelerating voltage.

### 3.4. Difference Scanning Calorimetry (DSC)

DSC studies were performed using a Mettler Toledo DSC822 (Mettler Toledo, Greifensee, Switzerland) calorimeter. The calorimeter was calibrated with indium and zinc. Samples weighing ≈ 5 mg were placed in an aluminum pan and sealed in a press. The degree of crystallinity of the obtained PHB+aPHA blends was calculated based on known melting enthalpy values. Equations (1) and (2) were utilized for these calculations.

A first heating scan was conducted from room temperature to 200 °C at 10 K/min to study the crystallinity of the received samples. The samples were then cooled down to −80 °C at a nominal rate of 10 K/min; however, due to a small instability of the liquid nitrogen cooler, the collected data were considered unreliable and were rejected. Finally, a second heating scan was conducted from −80 °C to 200 °C at 10 K/min to study glass transition and melting.

(1) $$x_{PHB}=\frac{\Delta H_{s}}{w_{PHB}\;\Delta H_{0}}$$

(2) $$x_{sample}=\frac{\Delta H_{s}}{\Delta H_{0}}$$

where

- $x_{PHB}$ is the degree of crystallinity of PHB, i.e., the mass of crystalline PHB of the total mass of PHB.
- $x_{sample}$ is the degree of crystallinity of the sample, i.e., the mass of crystalline PHB per unit mass of the sample.
- $\Delta H_{s}$ is the melting enthalpy of the sample, normalized to its mass.
- $\Delta H_{0}$ is the melting enthalpy of the 100% crystalline polymer, taken here as 146 J/g [36].

### 3.5. Thermograwimetry (TG)

Thermal stability was ascertained using the thermogravimetric analyzer NETZSCH TG 209F1 Libra (Netzsch, Selb, Germany) in an oxidizing and inert atmosphere at a heating rate 10 K/min. Samples weighing ≈ 5 mg were placed in corundum crucibles.

## 4. Results

### 4.1. Analysis of Materials Obtained in Step I

**Figure 1:**
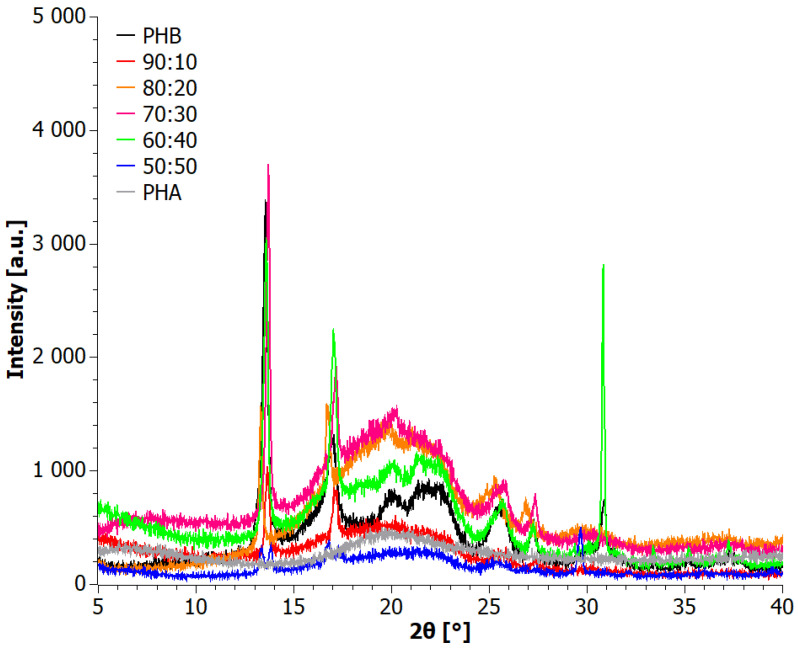
XRD patterns for solvent-casted PHB+aPHA blends.

The crystallinity of aPHA, PHB, and blends incorporating them was evaluated using XRD, and the results are presented in Figure 1. The XRD pattern of the aPHA sample exhibited a fully amorphous nature, displaying only a broad amorphous halo at 2*θ* = 20°. On the other hand, the diffraction pattern of PHB indicated its crystalline nature, with two prominent reflections observed at approximately 2*θ* = 13° and 17°, corresponding to (020) and (110) orthorhombic cells, respectively [37,38,39]. Additionally, a weaker broad peak around 2*θ* = 22.5°, attributed to the (111) reflections of the PHB α crystal, was detected. Other signals appearing near the 2*θ* angles of 25.5° and 27° have been described in the literature as corresponding to the planes (130) and (040) [37,38,39]. Notably, a diffraction signal assigned to the β form of PHB crystals was observed around 20°, indicating the development of both crystalline forms of PHB [37,38,39]. It is worth mentioning that the α form is the most common PHB crystal structure, which is typically formed under conventional melt or solution crystallization conditions [37,38,39]. Interestingly, despite the quite high content of the fully amorphous aPHA in the blends, their diffractograms showed high crystallinity retaining relatively strong crystalline peaks. Even more interesting is the fact that the sharpest peaks, i.e., better formed crystals, appeared at the intermediate concentrations of 60–70 wt.% PHB. This should be attributed to changes in the viscosity that probably promote mobility and thus crystal growth. As expected, with a further reduction of PHB content, the crystallinity dropped, and eventually for the 50:50 PHB+aPHA blend, the β form of PHB seemed to be absent.

**Figure 2:**
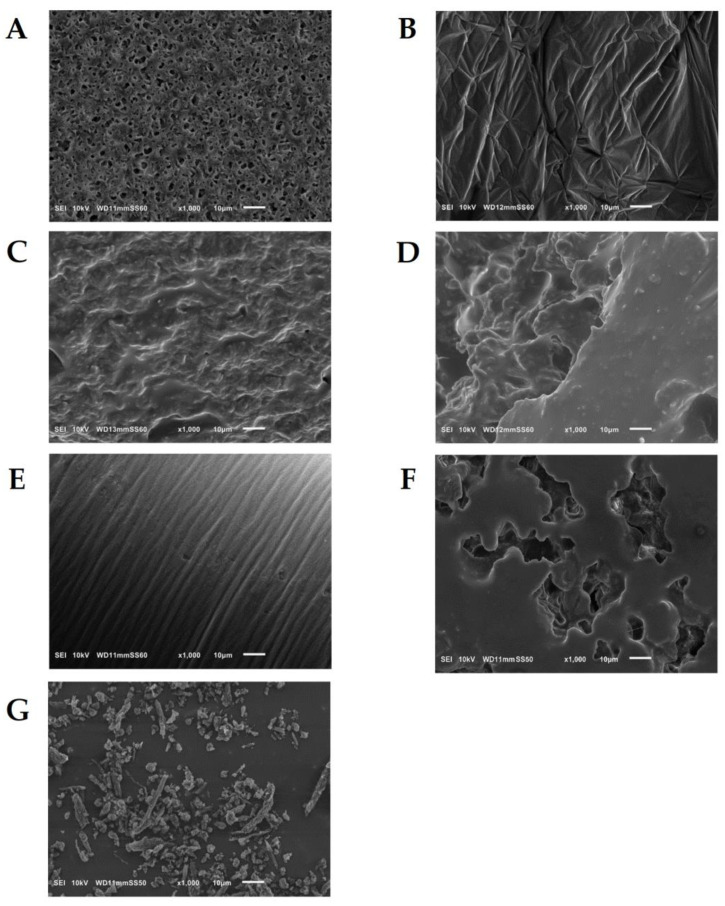
SEM images for solvent-casted PHB+aPHA blends and reference samples where (**A**) PHA; (**B**) PHB; (**C**) 90:10; (**D**) 80:20; (**E**) 70:30; (**F**) 60:40; (**G**) 50:50.

SEM images were captured to examine the morphology of the blends and reference samples. Figure 2 provides an overview of the surfaces discussed. The surface of the aPHA sample exhibited a multilayer cellular structure resembling open-cell foam. This unique structure of synthesized aPHA holds the potential for developing foaming PHA technology, enabling the production of highly flexible and insulating bio-materials. SEM analysis of the PHB sample confirmed the observations made during the materials’ preparation. The neat PHB sample demonstrated facile crystallization from the solvent, resulting in a continuous and processing-stable surface, making it favorable for applications such as membranes and thin films. However, the addition of 10% aPHA by weight disrupted this structure, resulting in a porous surface with significant heterogeneity. Some inclusions of aPHA were observed on the surface, interspersed within crystal phase of PHB. Similar patterns were observed for the 80:20 sample, although the size of the aPHA inclusions was approximately 5 μm. The SEM image indicated that the aPHA were enclosed within a continuous matrix of PHB throughout the volume of the blend. Additionally, SEM micrographs confirmed the findings of XRD analysis, demonstrating that even a small addition of aPHA to PHB significantly influenced the crystallization behavior of the dominant component. When exceeding the threshold of 30% mass content, aPHA altered the morphology of the blends, and no significant inclusions of this phase were observed. In contrast, for the 50:50 sample, both phases were observed to occur independently. Crystalline PHB was formed on the surface of amorphous PHA, with the distribution of crystallites being random. Notably, the dimensions of individual PHB crystallites reached up to 30 μm.

**Figure 3:**
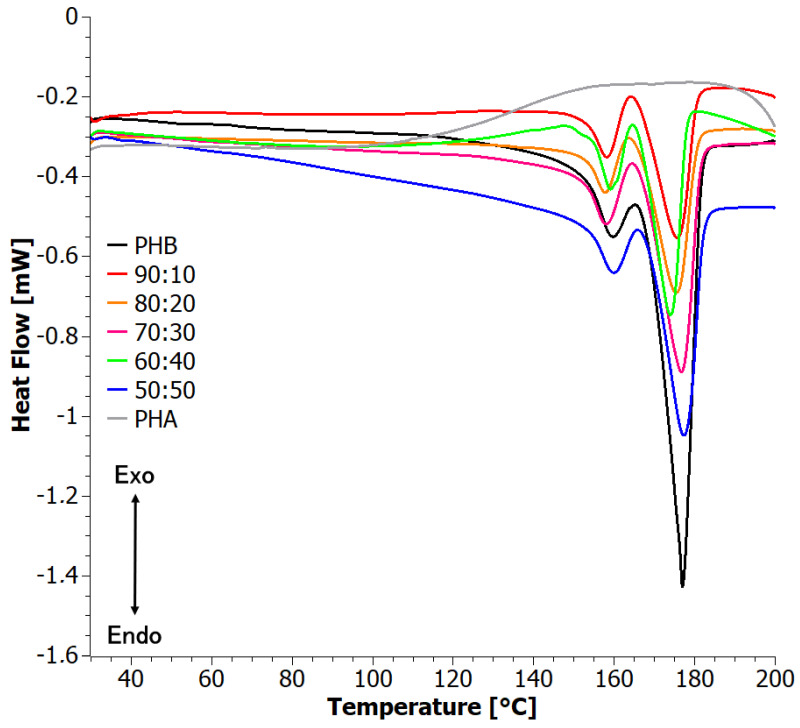
First heating DSC curves for solvent-casted PHB+aPHA blends.

The first heating DSC curves (Figure 3) of PHB and the blends showed two well-defined melting peaks, presumably due to the two different crystal forms of PHB, α and β, as observed also by XRD. With increasing PHA content on the blends, the temperature of the peaks showed only subtle, non-monotonic variations, indicating that blending with PHA does not significantly influence the quality/size of the formed PHB crystals [40]. On the other hand, the degree of crystallinity, both with respect to the blend mass and PHB mass, decreased at small PHA contents but then increased again. This should be attributed to two competing phenomena. On one hand, the addition of PHA introduced disorder to the system, but on the other hand, phase separation did occur, as observed by SEM, thus allowing for the ordering of the strongly crystalline PHB. Taking into account that crystallization was performed from solution, the process is very complex and depends on the interaction between three substances. This process is worth studying in future work.

**Figure 4:**
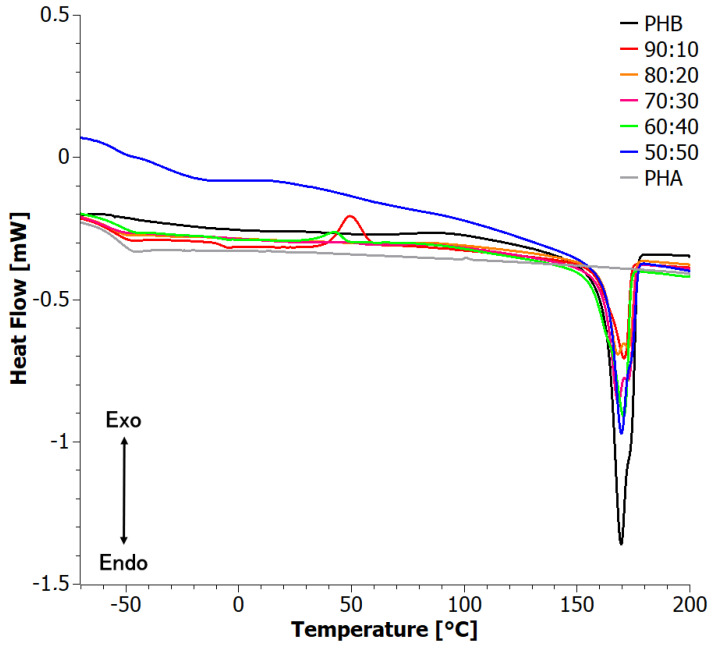
Second heating DSC curves for solvent-casted PHB+aPHA blends.

The second DSC heating curves (Figure 4) reflect crystallinity developed by cooling from the melt at a controlled rate. Instead of two well-defined peaks, a complex peak was present at intermediate temperatures, with the low-temperature component appearing at around 167 °C and the second one at around 173 °C. The location of the peaks did not vary significantly with composition, but their relative intensity showed strong and non-monotonic variations. This shows that crystallinity is very sensitive to the composition. It is interesting to note that for materials for which the high-temperature component is dominant, a cold crystallization exotherm was observed. Apparently, cold crystallization favors the development of the crystal form that corresponds to the high-temperature melting endotherm.

The degree of crystallinity developed by cooling was somewhat lower than that of the crystallinity developed from the solution (first run) (Table 1), presumably due to faster kinetics in the solution, which allows for better crystal growth. Like in the first run, at 10 wt.% PHA content, the degrees of crystallinity, with respect both to the blend and PHB mass, dropped significantly as compared to the neat PHB, but then the values increased again monotonically. This was possibly a result of the interplay between the disorder introduced by PHA and the changes in viscosity that control crystallization kinetics.

The step around −55 °C observed for PHA and the blends corresponded to the glass transition of the amorphous PHA. The glass transition of PHB is expected at around 0 °C [41]; however, no step was observed in the DSC curve of PHB in this region. This was a result of the quite high degree of crystallinity and the development of a significant amount of an amorphous fraction around crystallites, which is immobile and thus does not provide a glass transition (rigid amorphous fraction, RAF, [41,42]). A second glass transition, clearly associated with PHB, was observed only for the blends with 20 and 40 wt.% of PHA. This was correlated with the fact that crystallization had not been completed during cooling, as evidenced also by the presence of a cold crystallization peak at somewhat higher temperatures.

With increasing PHB content, the glass transition temperature of PHA seemed to migrate to lower values, in a non-monotonic manner (Table 1). This is a somewhat striking result, as blending with PHB, a polymer with a higher glass transition temperature around 0 °C [41], would rather cause the opposite trend. This behavior should then be associated with an increase in the free volume of the aPHA component.

**Figure 5:**
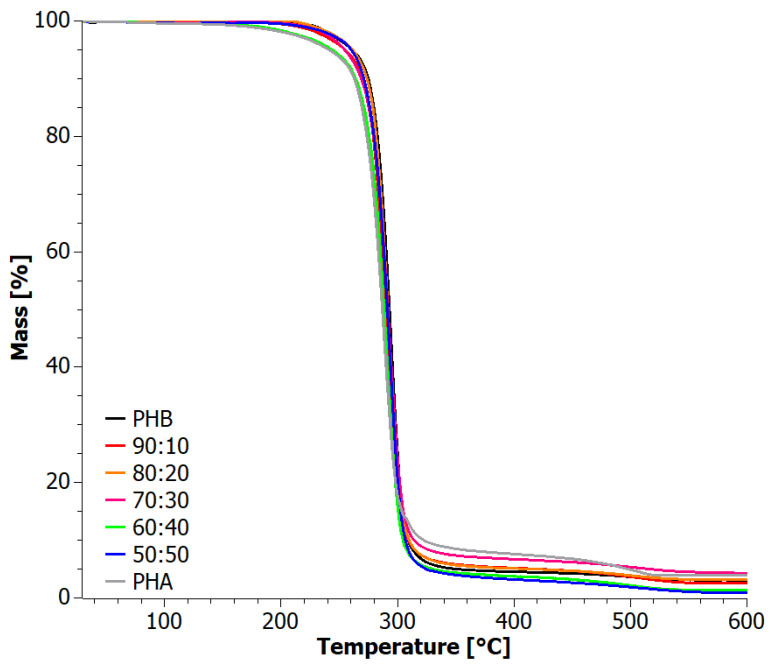
TG curves for solvent-casted PHB+aPHA blends, tested in an oxygen atmosphere.

Thermogravimetric measurements were conducted to further analyze the thermal stability of the blends (Figure 5). The thermo-oxidative degradation curves of the PHB+aPHA blends exhibited very similar decomposition patterns. The main decomposition step occurred at around 290 °C, while a very weak second decomposition step was visible at around 500 °C PHB, which demonstrated the highest thermal stability, with a maximum decomposition temperature of approximately 295 °C, consistent with the literature findings of El-Hadi et al. [43]. The TG curve of PHA indicated that the amorphous PHA sample exhibited the least thermal stability, displaying a two-stage decomposition process with a maximum of around 289 °C. The blends showed intermediate values for the temperature of the maximum decomposition rate (Table 2). Nevertheless, the loss in thermal stability by blending with PHA was minimal, and the materials were still suitable for applications at the same temperature conditions.

Additional thermogravimetric indices can be found in Table 2 for comparison. Comparing the mass loss trends of the blends subjected to heating under constant temperature increases in oxidizing and inert atmospheres, it was observed that they degraded similarly in the temperature range of 30–300 °C. Differences became apparent in the range of 300–510 °C, where a change in the degradation mechanism was observed.

**Figure 6:**
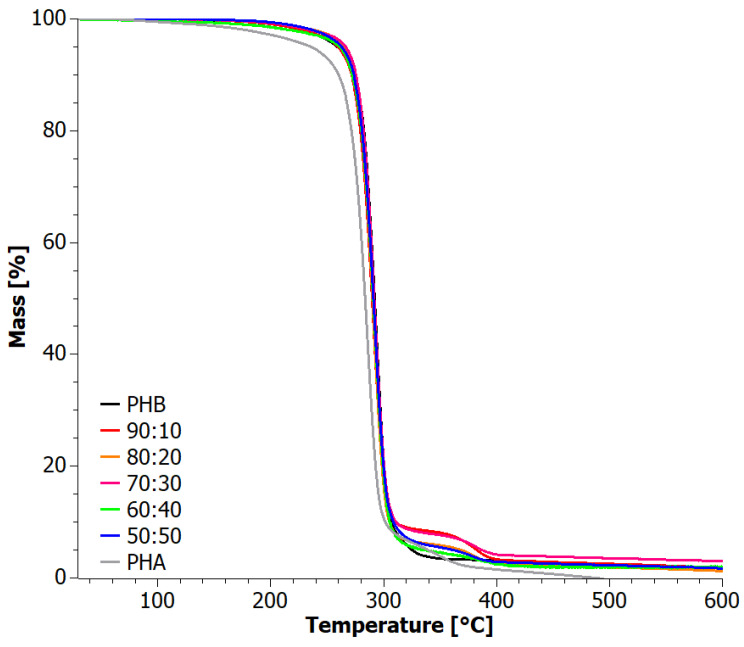
TG curves for solvent-casted PHB+aPHA blends, tested in an inert atmosphere.

The degradation curves of the PHB+aPHA blends under an inert atmosphere (Figure 6) exhibited a similar two-stage course. In the inert environment, the step around 500 °C was absent, but an additional stage of decomposition occurred at temperatures between 340 and 390 °C for PHA and the blends, reflecting that it is associated with the non-oxidative degradation of PHA. This resulted in a lower residue compared to thermal degradation under oxidizing conditions. Interestingly, this second degradation step migrated to higher temperatures with increasing PHB content (Table 2).

Based on the findings presented above, it can be concluded that blends with an excess of PHB relative to aPHA exhibit more favorable physicochemical properties and thermal processing characteristics. The presence of aPHA significantly influenced the thermal and morphological properties of the blends, particularly in compositions with substantial differences, such as the 90:10, 80:20, or 70:30 samples. This influence was evident in the rate of crystallization and the degree of crystallinity, which were prolonged and reduced, respectively. Blends with an excess of aPHA pose challenges or even limitations in their processing using conventional high-temperature methods.

However, when the mass fraction of PHB+aPHA was equalized, significant improvements were observed in various parameters, indicating the dominance of the PHB phase over the amorphous PHA phase. The degree of crystallinity increased, and the trend of thermal decomposition confirmed the shift towards the PHB phase. These improvements justify further research and development of PHB+aPHA blends with an excess of the PHB component.

### 4.2. Analysis of Materials Obtained in Step II

For high-temperature processing studies, blends with mass ratio PHB:aPHA at 60:40, 70:30, and 90:10 were prepared. The results obtained for materials prepared by solvent casting showed favorable properties for blends at these three mass ratios, and these blends were selected for further processing via extrusion. For comparison, pure PHB was also extruded.

**Figure 7:**
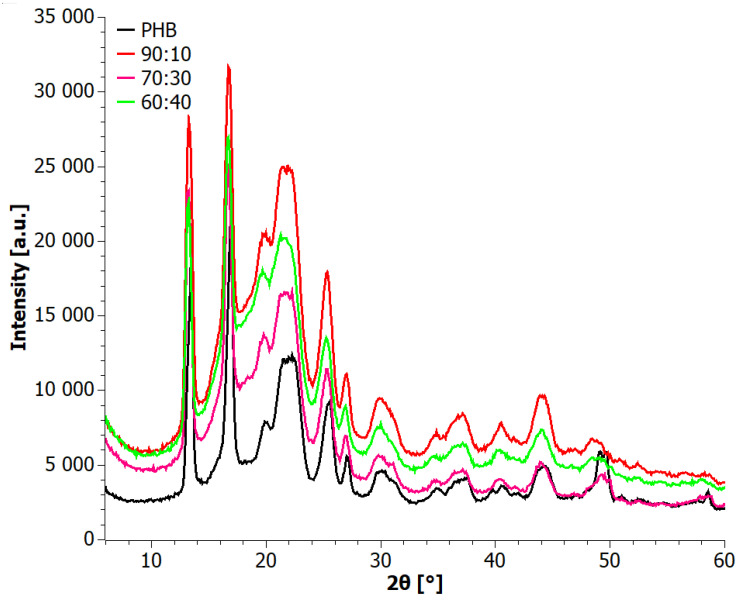
XRD patterns for extruded PHB+aPHA blends.

The diffractograms of the tested blends prepared in stage I revealed a predominantly crystalline nature of the materials. The most prevalent crystal structure observed was the α structure of PHB. Other reflections occurring around the 2*θ* angles of 25.5° and 27° corresponded to the (130) and (040) planes, respectively [38]. XRD diffractograms of the extruded materials (Step II) are shown in Figure 7. All samples exhibited a crystalline character. The characteristic reflections were observed at 2*θ* angles of 13°, 17°, 20°, 22.5°, and 27°, consistent with the XRD analysis of the blends obtained through the solvent casting method in stage I. The presence of these characteristic reflections confirmed the presence of both α and β forms of PHB crystals, as described in the literature [37,38,39] and earlier in the article at hand. Notably, the diffraction patterns after extrusion displayed a higher intensity of reflections compared to the tests conducted on blends obtained through solvent casting. This suggests an increased degree of crystallinity of the PHB phase in the blends after the extrusion process.

**Figure 8:**
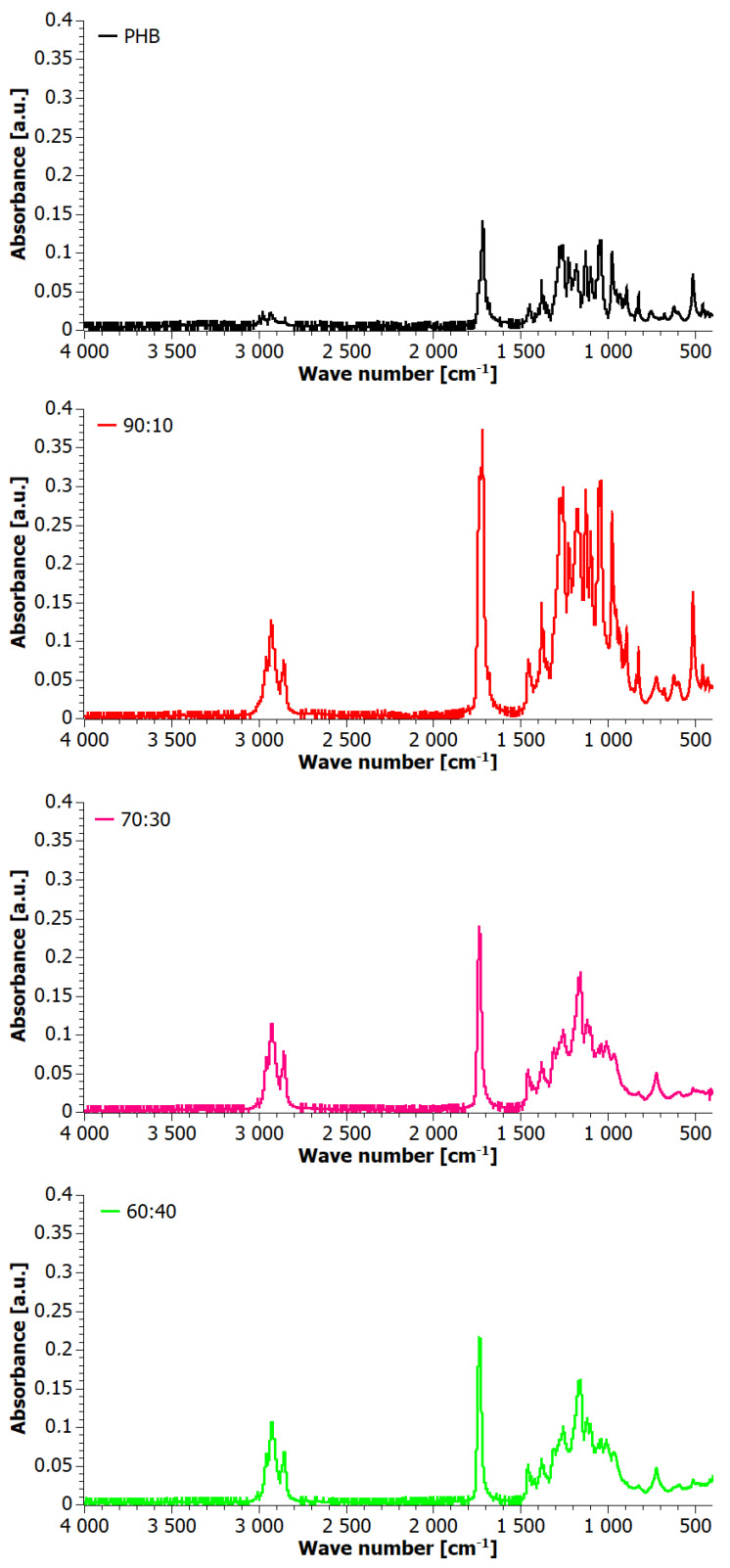
FTIR spectra for extruded PHB+aPHA blends.

To further analyze the structure of the blends after extrusion in stage II, the FTIR method was employed, and the results are shown in Figure 8. The FTIR spectra displayed absorption bands mainly characteristic of polyhydroxybutyrate, originating from C-H stretching vibrations in the range of 2916 cm^−1^. PHB showed two major C=O stretching bands at 1737 cm^−1^ and 1718 cm^−1^ in the amorphous and crystalline phases. In the range of 1300 cm^−1^–1000 cm^−1^, bands originating from C-O-C and C-C stretching, as well as CH deformation, were visible [44]. The spectra of the blends revealed changes, especially for 60:40 and 70:30 blends, evidencing a decrease in crystallinity of PHB due to its smaller share in the blend, which correlates with XRD patterns. This is visible through the decreasing intensity of the band at 1718 cm^−1^ in favor of the band at 1737 cm^−1^. Also, the intensity of the band at 1380 cm^−1^ resulting from CH_3_ symmetric deformation was reduced. Additionally, a decrease in the intensity of the bands assigned to crystalline states in PHB was observed, i.e., at 1276 cm^−1^ and 1227 cm^−1^ assigned to C-O-C stretching, and at 1054 cm^−1^ and 1043 cm^−1^ due to C-O stretching and C-CH_3_ stretching, respectively.

**Figure 9:**
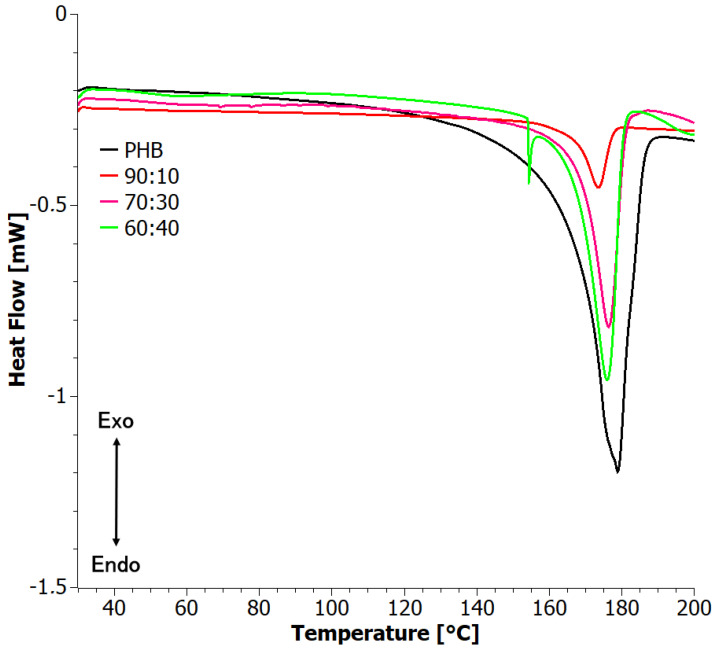
First heating DSC curves for extruded PHB+aPHA blends.

The first heating curves of the as-extruded blends showed a single peak at a temperature corresponding to the high-temperature component of the solvent-casted materials, indicating the formation of only one polymorph (Figure 9). This should be related to the many different conditions of crystallization, which in the case of extruded materials is practically quenching. The degrees of crystallinity were somewhat lower as compared to the solvent-casted materials (Table 3) due to the obvious restrictions in crystallization growth, i.e., restricted time of crystallization and higher viscosity of the system; however, we were still able to observe the phenomenon that small amounts of aPHA restrict crystallization of PHB but higher ones promote it.

**Figure 10:**
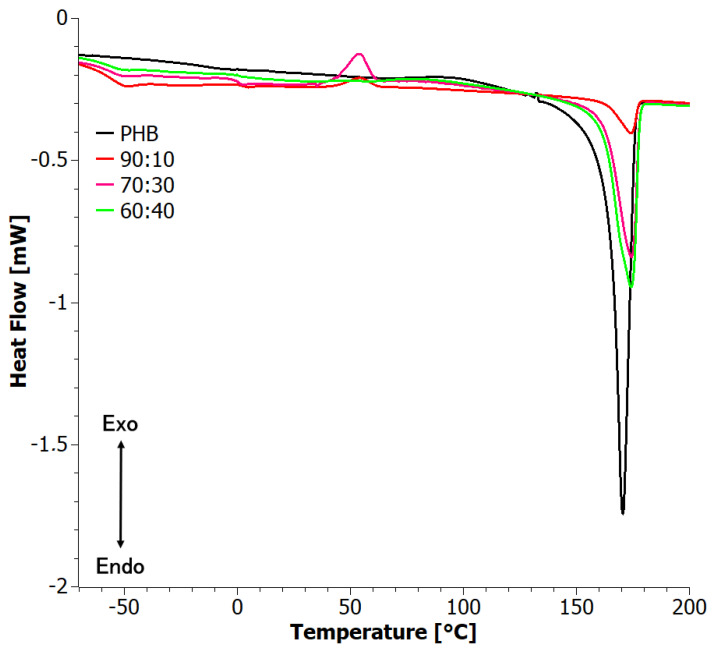
Second heating DSC curves for extruded PHB+aPHA blends.

The second heating curves are shown in Figure 10. Interestingly, two glass transition steps were clearly visible (Table 3). This indicates a strong phase separation of PHA from PHB in the materials. It is not clear at this point why this effect occurred only in the second run of the extruded materials and not in that of the solvent-casted ones; however, it should be related to subtle chemical changes at higher temperatures, or at residual solvent in solvent-casted ones.

At higher temperatures, the DSC curves indicated the presence of two crystalline forms of PHB, namely, α and β. Notably, the 90:10 blend showed a single peak, as was the case for the solvent-casted materials, indicating the presence of crystallites of only one type (β) in the material. In both the 90:10 and 60:40 blends, a cold crystallization peak was observed, suggesting the formation of ordered structures of a single type and a decrease in the content of the crystalline phase.

The degree of crystallinity of PHB (X_c_) and the degree of crystallinity of the obtained PHB+aPHA blends with mass ratios of 60:40, 70:30, and 90:10 were also calculated based on known melting enthalpy values (Table 3). The values are comparable to those of the solvent-casted materials, but the dependence on the composition was less clear. As was the case with the presence of second clear glass transition, this should be attributed to subtle physical or chemical changes during the exposure at high temperatures and pressures during the extrusion process or to residual solvent in the solvent-casted systems.

The reference sample, PHB, exhibited the highest degree of crystallinity among all the tested samples, reaching 55%. However, this material also displayed high brittleness, possibly due to the low nucleation density, which resulted in inter-spherulitic cracks in the presence of large spherulites. On the other hand, the blend with the lowest PHB content, specifically the 90:10 PHB:aPHA ratio, exhibited the lowest degree of crystallinity, which is a rather surprising result that is worth further investigation.

The DSC curves for the first heating exhibited a similar pattern for all samples. The melting peaks were determined at 175 °C for the 60:40 sample, 176 °C for the 70:30 sample, and the highest at 173 °C for the 90:10 sample. In the second temperature program (sample cooling), the onset of crystallization for the 60:40 sample occurred at around 47 °C, with a maximum crystallization peak at 60 °C. For the 70:30 and 90:10 blends, the crystallization peaks reached maximums of 58 °C and 59 °C, respectively. The second heating curve (Figure 10) revealed the presence of cold crystallization peaks, observed only in the 70:30 and 90:10 samples. The maximum cold crystallization peak for the 70:30 sample was at 53 °C, while the 90:10 sample exhibited a less pronounced cold crystallization peak at 54 °C. The resulting crystalline phase then melted in the temperature range of 155 °C to 180 °C, with a peak melting temperature of 174 °C for the 60:40 sample. The 70:30 sample had a melting peak at 174 °C, while the 90:10 sample exhibited a maximum melting peak at 174 °C as well.

Of the three blends tested, for the 90:10 and 60:40 samples, the method of obtaining them did not affect either the form of the crystallites or the effect of cold crystallization. However, for the 70:30 blends, high-temperature processing particularly affected the formation of the crystalline phase in the system.

**Figure 11:**
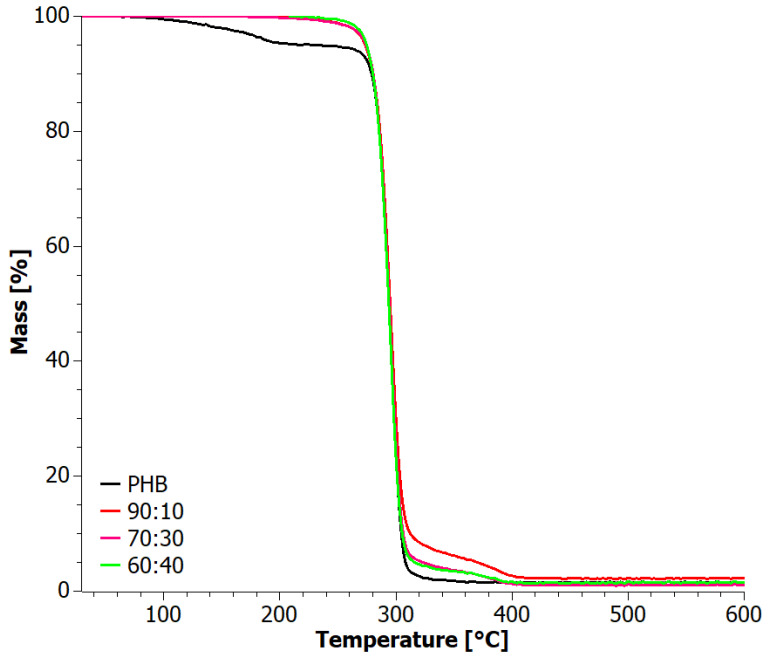
TG curves for extruded PHB+aPHA blends, tested in an inert atmosphere.

Thermogravimetric curves under inert gas conditions of the PHB+aPHA blends with mass ratios of 60:40, 70:30, and 90:10 after extrusion are displayed in Figure 11. A two-stage decomposition process was observed for all blends, as was the case with the solution blended systems, and with similar decomposition temperatures. Thermal degradation occurred similarly in all cases within the temperature range of 282 °C to 304 °C. The first stage of decomposition reached a maximum at around 296 °C, while the second stage occurred in the temperature range of 370–390 °C, with a maximum of 387 °C. The mass of the residue at 600 °C was very similar for PHB, 60:40, and 70:30 samples (Table 4). On the other hand, pure PHB showed a low-temperature decomposition step, below 200 °C, which likely indicates a compromise of the thermal stability during extrusion.

The above results show that the method of obtaining blends neither affected the subsequent nature of their decomposition route (two-step process) nor the range of decomposition temperatures and maximum melting points.

**Figure 12:**
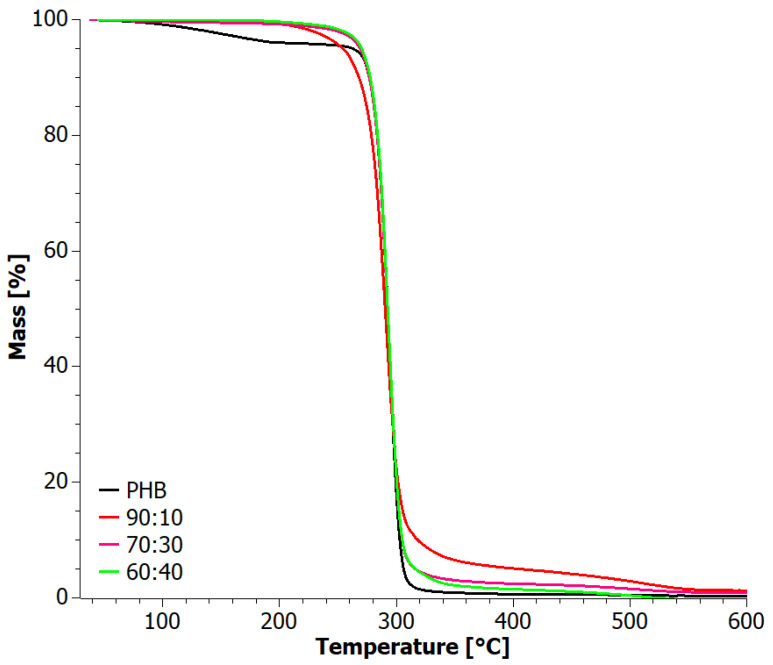
TG curves for extruded PHB+aPHA blends, tested in oxygen atmosphere.

Figure 12 shows the TG curves for samples tested under oxidizing conditions. The low-temperature decomposition step below 200 °C, observed at inert conditions for the pure PHB, was also present here. The best thermal stability at low decomposition temperatures (up to 300 °C) was exhibited by samples 60:40 and 70:30. Their thermograms practically overlapped. The 90:10 sample and the PHB sample, on the other hand, had significantly lower initial degradation temperature.

On the other hand, once T_max_ was exceeded, the trend reversed. During the second stage of decomposition, the order of charring of the samples can be ranked as follows: PHB > 60:40 > 70:30 > 90:10. This means that in extruded blends, as the proportion of aPHA increases, the susceptibility to sample charring increases.

## 5. Conclusions

Two biopolymers, PHB and aPHA, synthesized by fermentation using rapeseed oil hydrolysis products, were used to obtain PHB+aPHA blends. PHB was brittle, and aPHA was an amorphous viscous liquid with a yellow color. Therefore, it was decided to divide the process of obtaining blends into two stages: stage I—solvent casting, and stage II—the extrusion process. The results show that obtaining blends using one of the above-mentioned techniques is insufficient and they must occur together one after the other. Evidence is provided, for example, by differential scanning calorimetry (DSC) analysis, which revealed that the blends exhibited bimodal behavior indicating the presence of α and β crystalline forms of PHB. The degree of crystallinity varied with the conditions of crystallization (during extrusion, from solution, and by controlled heating from the melt). However, a consistent pattern was observed for the dependence of crystallinity of the PHB on the composition of the blends, namely, up to 20 wt% aPHA seemed to inhibit crystallization of PHB, but higher contents seemed to restore the crystallizability of PHB under the current conditions. This counterintuitive reversal should be connected with an enhancement of mobility. The blends showed a unique morphology, with the aPHA phase distributed mainly in the PHB phase. Thermogravimetric analysis showed similar decomposition patterns of the mixtures, with the decomposition process being a two-step process. X-ray diffraction analysis confirmed the crystalline nature of PHB and the amorphous nature of aPHA. SEM images revealed the morphology of the blends, showing the effect of aPHA content on their structure. PHB+aPHA blends subjected to the high-temperature extrusion process in the second step were characterized by more favorable physicochemical properties. The presence of aPHA significantly influenced the thermal and morphological properties of the mixtures, and the degree of crystallinity surprisingly increased with the increase in the aPHA content. Overall, the plasticization of PHB with aPHA expands the application potential of this promising group of biopolymers. Adding plasticizers to PHB blends may not be necessary because aPHA itself can perform this function under certain processing conditions.

## Figures and Tables

**Table 1:** Melting temperature (T_m_), melting enthalpy (ΔH_m_), glass transition temperature (T_g_), and degrees of crystallinity with respect to total blend mass (χ_Blend_) and PHB mass (χ_PHB_).

	First Heating					Second Heating					
Sample	T_m1_ (°C)	T_m2_ (°C)	ΔH (J/g)	χ_Blend_	χ_PHB_	T_g,PHB_ (°C)	T_g,PHA_ (°C)	T_m_ (°C)	ΔH_m_ (J/g)	χ_Blend_	χ_PHB_
PHB	159.5	176.9	68.6	0.47	0.47	---	---	169.1	63.7	0.44	0.44
90:10	158.2	175.7	25.9	0.18	0.20	−6.5	−55.2	171.1	18.6	0.13	0.14
80:20	157.8	175.5	25.7	0.18	0.22	---	−56.8	168.2	23.3	0.16	0.20
70:30	157.8	176.8	35.2	0.24	0.34	---	−58.8	168.4	32.3	0.22	0.32
60:40	159.3	174.0	30.4	0.21	0.35	−7.9	−51.8	170.2	29.2	0.20	0.33
50:50	159.7	177.3	38.3	0.26	0.52	---	−55.3	170.3	31.9	0.22	0.44
PHA	---	---	---	---	---	---	-54.0	---	---	---	---

**Table 2:** TG indices determined for PHB+aPHA blends based on Figure 5 and Figure 6.

Sample	T_Onset_ (°C)		T_Endset_ (°C)		DTG First Peak (°C)		DTG Second Peak (°C)		Residue (%)	
	Ox	Inert	Ox	Inert	Ox	Inert	Ox	Inert	Ox	Inert
PHA	271.6	269.4	299.6	295.1	289.1	286.1	509.1	348.0	3.89	0
50:50	277.6	278.6	303.6	302.1	293.5	292.2	---	376.8	0.85	1.69
60:40	274.3	279.6	301.2	307.1	291.3	292.2	---	382.4	1.40	2.00
70:30	276.6	279.3	304.2	302.1	293.7	292.6	---	383.8	4.27	2.99
80:20	277.7	277.0	303.9	300.9	293.1	291.5	---	383.8	3.22	1.28
90:10	275.5	276.3	302.9	300.7	291.7	291.1	---	381.2	2.66	1.78
PHB	289.6	279.7	304.8	303.5	294.8	292.9	---	---	3.09	1.72

Ox—oxygen atmosphere; Inert—inert atmosphere.

**Table 3:** DSC indicators of melting temperature (T_m_), melting enthalpy (ΔH_m_), and glass transition temperature (T_g_), and crystallinity with respect to total blend mass (χ_blend_) and PHB mass (χ_PHB_).

Sample	First Heating				Second Heating					
	T_m_ (°C]	ΔH_m_ (J/g)	χ_blend_	χ_PHB_	T_g,PHB_ (°C)	T_g,PHA_ (°C)	T_m_ (°C)	ΔH_m_ (J/g)	χ_blend_	χ_PHB_
90:10	167.2	7.3	0.05	0.06	1.0	−56.2	174.0	6.5	0.04	0.05
70:30	176.4	38.2	0.26	0.37	−0.1	−56.2	174.2	35.6	0.24	0.35
60:40	175.9	43.8	0.30	0.50	1.3	−56.5	174.1	45.0	0.31	0.51

**Table 4:** TG indices determined for PHB+aPHA blends based on Figure 11 and Figure 12.

Sample	T_Onset_ (°C)		T_Endset_ (°C)		DTG 1st Peak (°C)		DTG 2nd Peak (°C)		Residue (%)	
	Ox	Inert	Ox	Inert	Ox	Inert	Ox	Inert	Ox	Inert
60:40	281.3	282.4	303.6	304.1	293.2	296.0	509.6	387.8	0.0	1.5
70:30	280.7	282.7	303.7	304.6	293.9	296.1	493.3	387.5	0.9	1.1
90:10	275.4	283.0	303.3	305.1	291.6	296.0	508.8	387.6	1.3	2.3
PHB	282.7	284.4	303.0	304.7	149.5	138.6	294.0	296.3	0.3	1.6

Ox—oxygen atmosphere; Inert—inert atmosphere.

## Data Availability

The data are contained within the article.
